# Comparative profiling of skeletal muscle models reveals heterogeneity of transcriptome and metabolism

**DOI:** 10.1152/ajpcell.00540.2019

**Published:** 2019-12-11

**Authors:** Ahmed M. Abdelmoez, Laura Sardón Puig, Jonathon A. B. Smith, Brendan M. Gabriel, Mladen Savikj, Lucile Dollet, Alexander V. Chibalin, Anna Krook, Juleen R. Zierath, Nicolas J. Pillon

**Affiliations:** ^1^Department of Molecular Medicine and Surgery, Karolinska Institutet, Stockholm, Sweden; ^2^Department of Physiology and Pharmacology, Karolinska Institutet, Stockholm, Sweden

**Keywords:** C2C12, metabolism, skeletal muscle, transcriptomics

## Abstract

Rat L6, mouse C2C12, and primary human skeletal muscle cells (HSMCs) are commonly used to study biological processes in skeletal muscle, and experimental data on these models are abundant. However, consistently matched experimental data are scarce, and comparisons between the different cell types and adult tissue are problematic. We hypothesized that metabolic differences between these cellular models may be reflected at the mRNA level. Publicly available data sets were used to profile mRNA levels in myotubes and skeletal muscle tissues. L6, C2C12, and HSMC myotubes were assessed for proliferation, glucose uptake, glycogen synthesis, mitochondrial activity, and substrate oxidation, as well as the response to in vitro contraction. Transcriptomic profiling revealed that mRNA of genes coding for actin and myosin was enriched in C2C12, whereas L6 myotubes had the highest levels of genes encoding glucose transporters and the five complexes of the mitochondrial electron transport chain. Consistently, insulin-stimulated glucose uptake and oxidative capacity were greatest in L6 myotubes. Insulin-induced glycogen synthesis was highest in HSMCs, but C2C12 myotubes had higher baseline glucose oxidation. All models responded to electrical pulse stimulation-induced glucose uptake and gene expression but in a slightly different manner. Our analysis reveals a great degree of heterogeneity in the transcriptomic and metabolic profiles of L6, C2C12, or primary human myotubes. Based on these distinct signatures, we provide recommendations for the appropriate use of these models depending on scientific hypotheses and biological relevance.

## INTRODUCTION

Skeletal muscle is one of the largest organs in the body and plays an important role in locomotion and whole-body metabolic homeostasis. The formation of myofibers during myogenesis and muscle repair involves the activation of progenitor cells, which proliferate as mononuclear myoblasts and eventually fuse to form multinucleated myotubes (10). Adult skeletal muscle also comprises nerves, vasculature, smooth muscle cells, fibroblasts, and resident immune cells, which all contribute to the different functions fulfilled by muscle. This complex arrangement makes it difficult to decipher the specific contribution of muscle cells to physiological outcomes and limits the methods available to study intramyocellular processes in vivo (11). Molecular insights can, however, be obtained from in vitro models of skeletal muscle, such as immortalized C2C12 cells derived from thigh muscle of mice (55) and L6 cells isolated from cultures of thigh muscle of newborn rats (36). The L6 cell line has been extensively used in metabolic studies (17, 51, 53), and several modified clones have been developed, such as the L6-GLUT4myc cells that were engineered to express a labeled, insulin-responsive glucose transporter 4 (GLUT4) (53). Cultured primary myoblasts from human biopsies have also been developed (6) and are now widely used in metabolic studies.

Rat L6, mouse C2C12, and human primary muscle cells are the most commonly used cellular models to study skeletal muscle in vitro (1). Increasingly, researchers use primary human cells as a model system that has closer relevance to human health, but comparative studies of C2C12, L6, and primary human muscle cells are scarce. These models exhibit striking differences in response to diverse stimuli that influence substrate metabolism (25, 35, 37, 50), such that C2C12 cells are generally used to assess the effects of contraction/exercise-like stimuli (29, 47) and muscle atrophy (27, 45), and L6 cells and human primary myotubes are used for the study of metabolism and nutritional cues (20, 34, 51). Despite the vast amount of experimental data on their metabolic properties, the transcriptional variation between these models is unknown. We hypothesized that differences in the transcriptomic landscape may account for the diverse metabolic and contractile phenotypes associated with these models. To address this, we compared the transcriptional variation between adult skeletal muscle and cell cultures using publicly available data sets, and we associated gene-expression changes with the relative response of each cell/tissue type to measurements of proliferation, glucose uptake, mitochondrial activity, substrate oxidation, and response to in vitro contraction.

## METHODS

### 

#### Transcriptomic data acquisition and processing.

Publicly available data from myotubes and skeletal muscle tissues were selected from the Gene Expression Omnibus database (Supplemental Table S1; see https://doi.org/10.5281/zenodo.1246757). The variability in transcripts due to the use of different arrays was accounted for by including samples from both cells and tissues for each platform and using ranked statistics. Because human, rat, and mouse samples were processed using species-specific arrays, we cannot exclude the possibility that species-specific differences identified in our analysis may be due to interarray variability. We selected platforms in which both skeletal muscle cells and tissue samples for human (GPL570, GPL6244, GPL17586), rat (GPL1355, GPL22740, GPL14746), and mouse (GPL81, GPL1261, GPL17400) were available. Only samples corresponding to untreated, control conditions were kept for the analysis. Skeletal muscle tissue samples were selected from different anatomical locations so that the analysis included a mix of oxidative and glycolytic fibers. Raw files were downloaded, and robust multi-array normalization was performed in unison for all samples from the same platform. For each human Ensembl annotation, the rat and mouse orthologs were found using the R package BioMart, and the arrays were merged based on the human Ensembl annotation. The database was then aggregated according to the official human gene symbol. When multiple Ensembl annotations were found for a single gene symbol, an average was calculated. Quantile normalization, median centering, and statistics were performed using the R package limma. Gene ontology analysis was carried out in clusterProfiler (56) on genes that passed a false discovery rate < 0.05. Code for the analysis is available at https://github.com/NicoPillon/Muscle_Models_Profiling.

#### Culture and differentiation of rat L6 muscle cells.

Rat L6 muscle cells were obtained from the American Type Culture Collection and grown in minimum essential medium Eagle α modification (AMEM; 5.5 mM glucose; no. 22571; Gibco), supplemented with 100 U/mL penicillin, 100 µg/mL of streptomycin, and 0.25 µg/mL of amphotericin B (no. 15240; Gibco) and 10% fetal bovine serum (FBS; no. F7524; Sigma-Aldrich). Subculturing was done by trypsinization, and cells were diluted to 10^5^ cells/mL and seeded in plates. After ~2 days, when the cells reached 90% confluence, differentiation was induced by switching to AMEM, supplemented with 100 U/mL penicillin, 100 µg/mL streptomycin, and 0.25 µg/mL amphotericin B and 2% horse serum. Differentiation was monitored under the microscope, and cells were used for experiments after 4–6 days. The absence of mycoplasma contamination was routinely confirmed by PCR.

#### Culture and differentiation of mouse C2C12 muscle cells.

Mouse C2C12 muscle cells were obtained from the American Type Culture Collection (ATCC CRL-1772) and grown in AMEM, supplemented with 100 U/mL penicillin, 100 µg/mL of streptomycin, and 0.25 µg/mL of amphotericin B and 10% fetal bovine serum. Subculturing was done by trypsinization, and cells were diluted to 2 × 10^5^ cells/mL, and seeded in plates. A day after the cells reached 100% confluence, differentiation was induced by switching to AMEM, supplemented with 100 U/mL penicillin, 100 µg/mL of streptomycin, and 0.25 µg/mL of amphotericin B and 2% horse serum. Differentiation was monitored under the microscope, and cells were used for experiments after 6–8 days. The absence of mycoplasma contamination was routinely confirmed by PCR.

#### Culture and differentiation of primary human skeletal muscle cells.

Primary cells were isolated from vastus lateralis skeletal muscle biopsies derived from 12 healthy men (age: 50.4 ± 10 yr; body mass index: 22.9 ± 2.4 kg/m^2^) (2). The Ethical Committee at Karolinska Institutet approved protocols. Myoblasts were left to proliferate in growth medium (F12/DMEM, 25 mM glucose; no. 31331; Thermo Fisher Scientific), supplemented with 100 U/mL penicillin, 100 µg/mL of streptomycin, and 0.25 µg/mL of amphotericin B and 20% FBS. Cells were used between *passage 6* and *9*. Differentiation to myotubes was induced in DMEM with GlutaMAX (no. 31966; Gibco) containing 20% medium 199 (no. 31150; Gibco), HEPES buffer (0.02 M; no. 15630; Gibco), zinc sulfate (0.03 μg/mL), vitamin B_12_ (1.4 μg/mL; Sigma-Aldrich), insulin (10 μg/mL; Actrapid; Novo Nordisk), and apo-transferrin (100 μg/mL; no. T100-5; BBI Solutions). After 4 days, cells were switched to postfusion medium containing DMEM/M199, HEPES, zinc sulfate, vitamin B_12_, and 2% FBS for 2 days. Cells were used for experiments 6–10 days after the initiation of differentiation. Differentiation was monitored under the microscope, and the absence of mycoplasma contamination was routinely confirmed by PCR.

#### Proliferation.

Myoblasts were seeded in 25 cm^2^ flasks and left to adhere and stabilize for 24 h. Five pictures of randomly chosen fields were taken every 24 h for 72 h on an inverted light microscope. Measurement of the surface occupied by cells was done using ImageJ (NIH, Bethesda, MD). Script is available on request. Results are the average of 5 independent experiments for C2C12, 5 independent experiments for L6 and 7 replicates from 7 individuals donors for HSMC.

#### Bromodeoxyuridine incorporation.

L6, C2C12, and primary human myoblasts were seeded in 96-well plates at densities of 10,000, 5,000, and 2,500 cells/well, respectively. Cells were then incubated with 10 µM bromodeoxyuridine for 12 h or 24 h, and the incorporation was measured according to the manufacturer’s protocol (BrdU Cell Proliferation Assay Kit; no. 6813; Cell Signaling Technology). Results are the average of 6 independent experiments for C2C12, 6 independent experiments for L6 and 6 replicates from 3 individuals donors for HSMC.

#### Immunocytochemistry.

Myotubes were washed with PBS, fixed in 4% paraformaldehyde for 10 min, and quenched with 0.1 M glycine for 10 min at room temperature. After membrane permeabilization with 0.1% Triton X-100 for 3 min, cells were washed with PBS, blocked with 1% BSA for 15 min at room temperature, and incubated with anti-myosin (MYH1/2; sc-53088; Santa Cruz Biotechnology) or anti-desmin (ab15200; Abcam) antibodies (1:250) overnight at 4°C with gentle rocking. Cells were next washed with PBS and incubated with Alexa Fluor 594 goat anti-mouse (A-11005) or Alexa Fluor 488 goat anti-rabbit (A-11008) secondary antibodies (1:500; Thermo Fisher Scientific), respectively, for 45 min in the dark at room temperature. Images were acquired using 20× magnification on a Zeiss Axio Vert.A1 inverted fluorescent microscope, equipped with ZEN software (Carl Zeiss Microscopy GmbH, Jena, Germany).

#### Lactate measurement.

Lactate was measured in supernatant medium using a colorimetric assay, based on the conversion of lactate to pyruvate by lactate dehydrogenase (4). Assay buffer contained Tris (50 mM, pH 8), NAD (7.5 mM), *N*-methylphenazonium methyl sulfate (250 µM), p-iodonitrotetrazolium violet (500 µM), and lactate dehydrogenase (4 U/mL). Cell supernatant was filtered through 3 kDa filters and 50 μL of sample were mixed with 150 µL of assay buffer, incubated for 20 min; and absorbance was read at 490 nm. Results were normalized to the protein content measured after lysis of the cells with 0.03% SDS (BCA Protein Assay Kit; no. 23225; Thermo Fisher Scientific, Rockford, IL). Results are the average of 5 independent experiments for C2C12, 5 independent experiments for L6 and 5 replicates from 4 individual donors for HSMC.

#### Glucose uptake.

After 2–4 h serum starvation in low-glucose DMEM (5.5 mM glucose; no. 21885; Gibco), myotubes were incubated in the absence or presence of insulin (100 nmol/L) for 20 min for L6 and C2C12 and 60 min for primary human myotubes. Glucose uptake was measured in glucose- and serum-free DMEM (no. 11966; Gibco) by adding 2-[1,2-^3^H]deoxy-d-glucose (1 mCi/mL and 80.7 Ci/mmol; MT911; Moravek) and 10 μmol/L unlabeled 2-deoxy-d-glucose for 15 min. Cell monolayers were washed with ice-cold PBS and lysed in 1 mL 0.03% SDS. The cell lysate (0.5 mL) was counted in a liquid scintillation counter (WinSpectral 1414; Wallac), and protein content was measured for normalization of results (BCA Protein Assay Kit; no. 23225; Thermo Fisher Scientific, Rockford, IL). Results are the average of 11 independent experiments for C2C12, 13 independent experiments for L6 and 11 replicates from 11 individual donors for HSMC.

#### Electrical pulse stimulation.

Differentiated myotubes were subjected to electrical pulse stimulation (EPS; C-Pace EP; Ionoptix, MA) at 40 V, 2 ms, 1 Hz during 3 h in low-glucose DMEM. RNA was extracted immediately and analyzed by quantitative PCR (qPCR), as described below. For glucose uptake, after 2 h stimulation, a solution of 1 mCi/mL 2-[1,2-^3^H]deoxy-d-glucose and 10 μmol/L unlabeled 2-deoxy-d-glucose was added to the medium. Glucose uptake was measured during the last hour of EPS. Cells were washed, lysed, and subjected to scintillation counting, as described above. Results are the average of 6 independent experiments for C2C12, 7 independent experiments for L6 and 7 replicates from 7 individual donors for HSMC.

#### Glycogen synthesis.

After 2–4 h serum starvation in low-glucose DMEM, myotubes were treated with 100  nM insulin for 30 min before adding 1 μL d-[U-^14^C] glucose (1  mCi/mL and 250  mCi/mmol; no. NEC042B005MC; PerkinElmer, Waltham, MA) for the last 90  min. Cell monolayers were washed with ice-cold PBS and lysed in 0.5  mL 0.03% SDS. The cell lysate (0.4  mL) was transferred to 2 mL tubes, and 0.1  mL (2  mg/sample) carrier glycogen (no. G0885; Merck) was added. The remaining cell suspension was used for protein concentration determination using the Pierce BCA Protein Assay Kit. The samples were heated to 99°C for 1 h. Glycogen was precipitated by addition of 95% ethanol and incubated overnight at −20°C. Glycogen pellets were collected by centrifugation for 15  min at 10, 000  *g*, washed once with 70% ethanol, and resuspended in 0.3  mL distilled water. [^14^C]-labeled glycogen was counted in a liquid scintillation counter and normalized to protein content. Results are the average of 9 independent experiments for C2C12, 8 independent experiments for L6 and 12 replicates from 12 individual donors for HSMC.

#### Fatty acid oxidation.

Myotubes were serum starved for 1 h in DMEM (5.5 mM glucose) and then incubated for 6 h in 1  mL low-glucose DMEM containing 25  μM of palmitate, including 0.078  μM [9,10-^3^H(N)] palmitate (5  mCi/mL and 53.7  Ci/mmol; NET043005MC; PerkinElmer) and 0.04% BSA. Supernatant was collected and incubated with 0.8  mL of charcoal buffer (10% activated charcoal in 0.02  M Tris-HCl buffer at pH 7.5) and shaken for 30  min. Samples were centrifuged (13,000 *g*, 15  min), and 0.2 mL of the supernatant was counted in a liquid scintillation counter. Cells were harvested in 0.03% SDS for protein content determination using the bicinchoninic acid (BCA) protein assay. Results are the average of 11 independent experiments for C2C12, 17 independent experiments for L6 and 37 replicates from 12 individual donors for HSMC.

#### Glucose oxidation.

Myotubes were serum starved for 1–2 h in DMEM (5.5 mM glucose), and then 1 mL serum-free DMEM containing 5.5 mM glucose and 2 mCi/mL d-[U-^14^C] glucose in the absence or presence of 0.5 µM carbonyl cyanide-4-(trifluoromethoxy) phenylhydrazone (FCCP) was added to each well. A small, empty cup was carefully added on the medium in each well before sealing the wells with a plastic seal and incubating them for 4 h at 37°C in 0% CO_2_. [^14^C]-CO_2_ was then released from the medium by injecting 150 μL 2 M HCl to the medium and captured by adding 300 μL 2 M NaOH to the cup and incubating the plates for 1 h at 37°C in 0% CO_2_. The [^14^C]-CO_2_ in 300 μL NaOH was counted using a liquid scintillation counter. Cells were harvested in 0.4 mL 0.5 M NaOH, pH was neutralized with 0.1 mL 2 M HCl, and protein content was determined by a BCA Protein Assay. Results are the average of 6 independent experiments for C2C12, 8 independent experiments for L6 and 19 replicates from 12 individual donors for HSMC.

#### Oxygen consumption and extracellular acidification rate (Seahorse).

To assess mitochondrial function of myotubes, cells were subjected to a Seahorse XF Mito Stress Test using the manufacturer’s instructions (Agilent, Santa Clara, CA). The Seahorse assay media was XF Base Media (no. 102353; Agilent, Santa Clara, CA), supplemented with 10 mM glucose, 1 mM sodium pyruvate, 2 mM glutamine, and pH adjusted to 7.4. Cells were seeded at 30,000 cells/well (20,000 cells/well for L6 and C2C12) in 24-well, assay-specific plates. Wells were washed, and differentiation media were added within 18 h of seeding. After differentiation, oxygen consumption rates (OCRs) and extracellular acidification rates (ECARs) were measured at three time points under unstimulated conditions and then after treatment with 1 µM oligomycin, 2 µM FCCP, and 0.75 µM rotenone/antimycin A. Results are the average of 7 independent experiments for C2C12, 8 independent experiments for L6 and 13 replicates from 8 individual donors for HSMC.

#### RNA extraction and analysis.

Cultured muscle cells were lysed, and RNA was extracted using the E.Z.N.A. Total RNA Kit (Omega Bio-tek, Norcross, GA), and concentration was determined through spectrophotometry. All equipment, software, and reagents for performing the reverse transcription and qPCR were from Thermo Fisher Scientific. cDNA synthesis was performed from ~0.1–1 µg of RNA using random hexamers and the High Capacity cDNA Reverse Transcription Kit, according to the manufacturer’s instructions. qPCR was performed on a StepOnePlus system. Probe sequences are available on request. Relative gene expression was calculated by the comparative threshold cycle (ΔΔCt) method using *18S*, *B2M*, or *TBP* as housekeeping genes. Results are the average of 6 independent experiments for C2C12, 6 independent experiments for L6 and 5 replicates from 5 individual donors for HSMC.

#### Statistics.

Analyses were performed using either R 3.5.2 (www.r-project.org) or GraphPad Prism 8.1 software (GraphPad Software Inc.). Normality was verified using the Shapiro-Wilk test. When data were normally distributed, ANOVA with Tukey’s multiple comparison was used. For data not normally distributed, a Kruskal-Wallis test with Dunn’s multiple comparison was used. Sample size and statistical tests are described in figure captions.

## RESULTS

### 

#### Transcriptomic differences between mouse, rat, and human skeletal muscle myotubes and tissues.

**Fig. 1.**
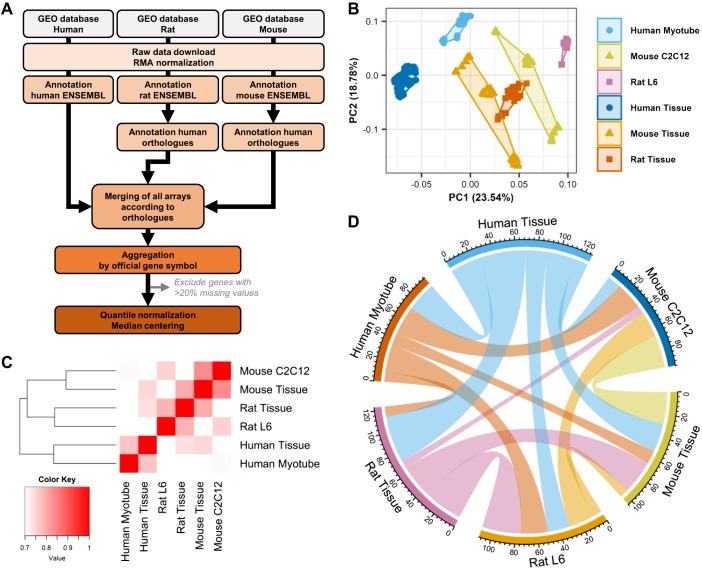
Transcriptomic profiling of rat L6, mouse C2C12, and human primary myotubes. Publicly available data sets for mouse, rat, and human skeletal muscle tissue and cell models were downloaded from the Gene Expression Omnibus (GEO) repository. *A*: raw data were processed, normalized, and annotated according to the official human gene ortholog nomenclature. *B*: principal component (PC) analysis demonstrated a clear segregation of all samples from each other. *C*: correlation matrix comparing all transcripts from one model to the other five. *D*: chord diagram presenting the overlap of the top 100 expressed genes in each model. The thickness of a band is proportional to the number of common genes between 2 models. RMA, robust multi-array.

**Fig. 2.**
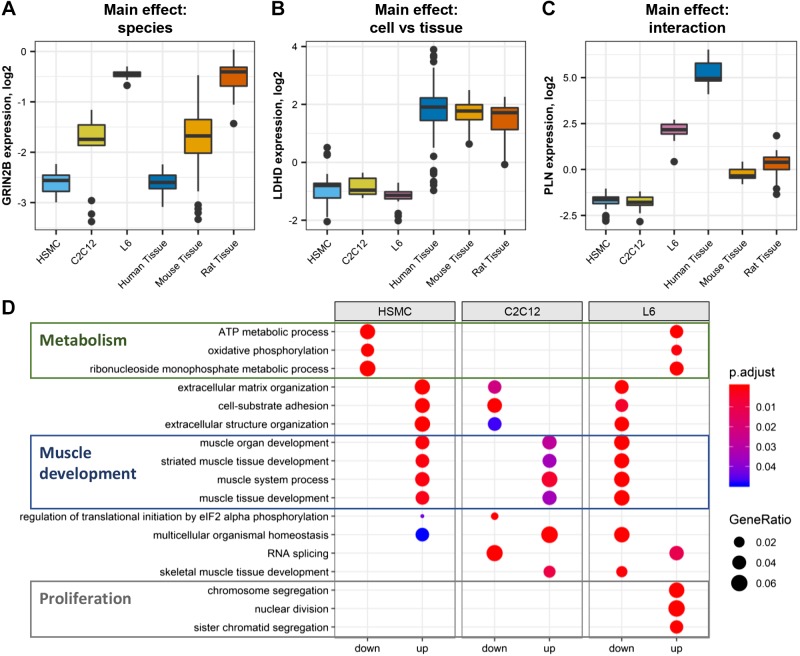
Gene enrichment in L6, C2C12, and human primary myotubes. *A*: example of a gene (*GRIN2B*) exhibiting species differences. *B*: example of a gene (*LDHD*) differentially expressed in cells compared with skeletal muscle tissue. *C*: example of a gene (*PLN*) which expression is affected by both species and cell/tissue sample. *D*: gene ontology enrichment of genes differentially expressed in one model compared with the other two (false discovery rate < 0.05). Gene ratio is the number of significantly different genes divided by the total number of genes associated with a specific pathway. eIF2, eukaryotic translation initiation factor 2; HSMC, human skeletal muscle cells; p.adjust, adjusted *P* value.

Public databases were mined for transcriptomic studies of mouse C2C12, rat L6, and human primary myotubes, as well as skeletal muscle tissue samples collected from the same transcriptomic platforms (Supplemental Table S1; see https://doi.org/10.5281/zenodo.1246757). After quality control, normalization, and annotation with the official human gene names (Fig. 1*A*), we observed a clear clustering of cell models and skeletal muscle tissues away from each other (Fig. 1*B*). The best correlations of transcript expression were observed between cells and tissues from the same species (e.g., mouse tissue vs. C2C12), suggesting important species-specific differences in skeletal muscle mRNA composition (Fig. 1*C*). Significant correlations emerged when comparing cell models with each other or with tissues from different species, highlighting differences between in vivo and in vitro models. When comparing the top 100 expressed genes, we observed a significant overlap between models (Fig. 1*D*). Overall, we found that genes expressed in the different cells and tissue models clustered into three categories: genes similarly expressed in cells and adult tissues but differentially expressed in mouse, rat, and human species, such as the glutamate ionotropic receptor *N*-methyl-d-aspartate type 2B (*GRIN2B*; Fig. 2*A*); genes expressed similarly across species but different in cells compared with adult tissue, such as the D isoform of lactate dehydrogenase (*LDHD*; Fig. 2*B*); and genes both different in cells compared with tissue and across species, such as phospholamban (*PLN*; Fig. 2*C*).

Gene ontology enrichment comparing the three cellular models revealed that rat L6 cells were enriched with pathways related to metabolism and proliferation, but genes related to muscle function and contraction were lowly expressed compared with other cell models (Fig. 2*D*). In contrast, primary human cells [human skeletal muscle cells (HSMCs)] had high levels of genes related to muscle development but a limited number of genes related to metabolic pathways. Mouse C2C12 cells were not particularly rich in metabolism-related genes but had increased levels of muscle-related pathways compared with L6 and HSMC cells. Collectively, our transcriptomic study reveals that the selective gene profiles of C2C12, L6 and HSMC cells in vitro can be attributed to both species differences and model-specific uniqueness, potentially affecting the response of the cells to proliferation, contraction, and metabolic stimuli.

#### Proliferative differences.

**Fig. 3.**
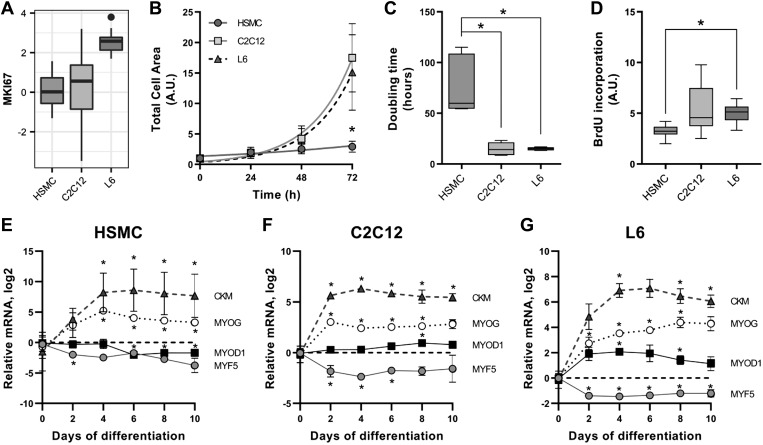
Proliferation rates of L6, C2C12, and human primary myotubes. *A*: mRNA expression of the proliferation marker *MKI67*. *B*: total area covered by cells during 3 days of proliferation. Quantification of images was done with ImageJ, as described in methods. Mean ± SE, Exponential (Malthusian) growth fit; *n* totals are as follows: HSMC: *n* = 7 replicates from 7 independent donors, C2C12: *n* = 5 independent experiments, L6: *n* = 5 independent experiments, *P < 0.05 in human skeletal muscle cells (HSMC) compared with L6 and C2C12. *C*: doubling time calculated from the proliferation curves. Box-and-whisker Tukey plot; *n* totals are as follows: HSMC: *n* = 7 replicates from 7 independent donors, C2C12: *n* = 5 independent experiments, L6: *n* = 5 independent experiments, Kruskal-Wallis test with Dunn’s multiple comparison, *P < 0.05. *D*: BrdU incorporation into DNA measured as described in methods. Box-and-whisker Tukey plot; *n* totals are as follows: HSMC: *n* = 6 replicates from 3 independent donors, C2C12: *n* = 6 independent experiments, L6: *n* = 6 independent experiments, one-way ANOVA with Tukey’s multiple comparison, *P < 0.05. *E–G*: differentiation time course of mRNA expression of skeletal muscle markers creatine kinase (*CKM*), myogenin (*MYOG*), myogenic differentiation 1 (*MYOD1*), and myogenic factor 5 (*MYF5*). Mean ± SE, Dot plot; *n* totals are as follows: HSMC: *n* = 5 replicates from 5 independent donors, C2C12: *n* = 4 independent experiments, L6: *n* = 4 independent experiments, paired one-way ANOVA with Dunnett’s multiple testing, *P < 0.05 compared with undifferentiated cells. A.U., arbitrary units.

Gene ontology analysis suggested that L6 cells might be more proliferative than C2C12 and HSMC cells, which was confirmed by increased expression of the proliferation marker *MKI67* in L6 myotubes (Fig. 3*A*). Quantification of the total cell area of myoblasts evaluated every 24 h for 72 h revealed that L6 and C2C12 myoblasts proliferate much faster than HSMCs (Fig. 3*B*). The average doubling time calculated from an exponential (Malthusian) growth fit was 76 ± 26 h for HSMC, 14 ± 6 h for C2C12, and 15 ± 2 h for L6 myoblasts (Fig. 3*C*). Correspondingly, the incorporation of bromodeoxyuridine was higher in L6 versus HSMC (Fig. 3*D*).

A differentiation time course was established for each model. HSMC, C2C12, and L6 reached maximal differentiation after 4 days in culture (Fig. 3, *E*–*G*). The differential status of HSMC and C2C12 remained stable for up to 10 days in culture, but L6 myotubes showed a decrease in myogenic differentiation 1 (*MYOD1*) and creatine kinase M type (*CKM*) after 6 days in culture (Fig. 3*G*). For subsequent analysis, cells were used at similar differentiation states.

#### Glucose uptake and glycogen synthesis.

**Fig. 4.**
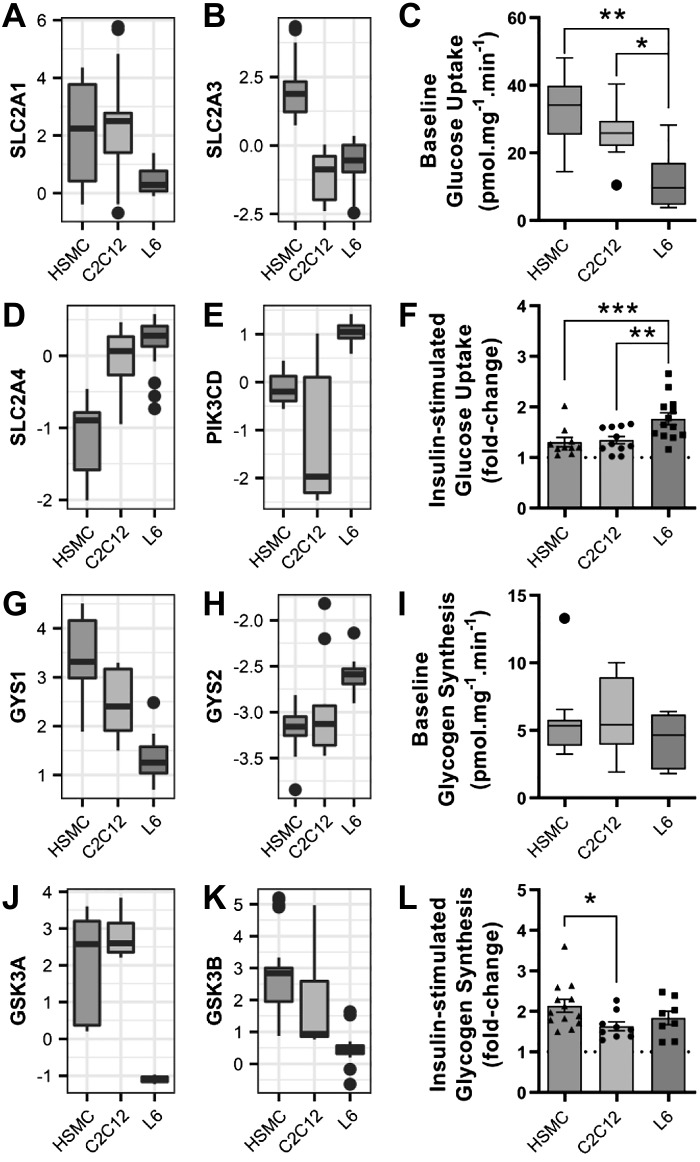
Insulin-induced glucose uptake and glycogen synthesis in L6, C2C12, and human primary myotubes. *A* and *B*: mRNA expression of glucose transporter 1 and 3 (*SLC2A1* and *SLC2A3*). *C*: basal glucose uptake, as measured using 2-[1,2-^3^H]deoxy-d-glucose. Box-and-whisker Tukey plot; *n* totals are as follows: HSMC: *n* = 11 replicates from 11 independent donors, C2C12: *n* = 11 independent experiments, L6: *n* = 13 independent experiments, one-way ANOVA with Tukey’s multiple comparison. *D* and *E*: mRNA expression of glucose transporter 4 (*SLC2A4*) and the delta subunit of phosphatidylinositol 3-kinase (*PIK3CD*). *F*: insulin-stimulated glucose uptake, as measured using 2-[1,2-^3^H]deoxy-d-glucose. Means ± SE and individual data points: *n* totals are as follows: HSMC: *n* = 11 replicates from 11 independent donors, C2C12: *n* = 11 independent experiments, L6: *n* = 13 independent experiments, one-way ANOVA with Tukey’s multiple comparison. *G* and *H*: glycogen synthase (GS) mRNA expression [GS 1 isoform (*GYS1*) and GS 2 isoform (*GYS2*)]. *I*: basal glycogen synthesis, as measured by the incorporation of [^14^C]glucose into glycogen. Box-and-whisker Tukey plot; *n* totals are as follows: HSMC: *n* = 12 replicates from 12 independent donors, C2C12: *n* = 9 independent experiments, L6: *n* = 8 independent experiments, Kruskal-Wallis test with Dunn’s multiple comparison. *J* and *K*: glycogen synthase kinase 3 (*GSK3*) mRNA expression. *L*: insulin-stimulated glycogen synthesis as measured by the incorporation of [^14^C]glucose into glycogen. Means ± SE and individual data points; *n* totals are as follows: HSMC: *n* = 12 replicates from 12 independent donors, C2C12: *n* = 9 independent experiments, L6: *n* = 8 independent experiments, one-way ANOVA with Tukey’s multiple comparison, *P < 0.05, **P < 0.01, ***P < 0.001. HSMC, human skeletal muscle cells.

Insulin-induced glucose uptake into skeletal muscle is fundamental for the regulation of postprandial glycemia. Gradient-driven baseline glucose uptake in skeletal muscle is facilitated by GLUT1 (*SLC2A1*) in adult tissue and GLUT3 (*SLC2A3*) in embryonic muscle (43). L6 myotubes expressed relatively low levels of *SLC2A1* (Fig. 4*A*), while HSMCs had high expression of *SLC2A3* (Fig. 4*B*). This functionally translated into L6 myotubes having the lowest baseline glucose uptake (Fig. 4*C*). GLUT4 (*SLC2A4*) is the only insulin-dependent glucose transporter present in skeletal muscle and is translocated to the plasma membrane upon activation of the canonical insulin signaling cascade (22). L6 myotubes exhibited the highest levels of *SLC2A4* (Fig. 4*D*), and the delta subunit of phosphatidylinositol-4,5-bisphosphate 3-kinase (*PIK3CD*; Fig. 4*E*), suggesting that they are more responsive to insulin. Indeed, maximal insulin-stimulated glucose uptake in L6 cells was increased by 1.8-fold (Fig. 4*F*), and this effect was higher than C2C12 cells (1.3-fold) and HSMCs (1.2-fold).

Skeletal muscle stores glucose in the form of glycogen during feeding periods in response to the activation of glycogen synthase (GS). Adult skeletal muscle mainly expresses the glycogen synthase 1 isoform (*GYS1*), while liver relies on glycogen synthase 2 (*GYS2*) (14). HSMC cells had the highest expression of *GYS1* (Fig. 4*G*), whereas *GYS2* content was low in all three cell models (Fig. 4*H*). Baseline glycogen synthesis did not differ between the cell models (Fig. 4*I*).

Glycogen synthase, a key enzyme in glycogen synthesis, is activated by the allosteric stimulator glucose-6-phosphate (7) and by dephosphorylation via the protein kinase B (PKB/Akt) and glycogen synthase kinase 3 (GSK3) pathway (12). L6 myotubes had the lowest expression of both the alpha (*GSK3A*; Fig. 4*J*) and beta (*GSK3B*; Fig. 4*K*) isoforms of GSK3, but the insulin-stimulated glycogen synthesis in L6 cells (1.8-fold) was not different from the other myotube models (Fig. 4*L*). HSMC cells had the greatest fold increase in insulin-stimulated glycogen synthesis (2.1-fold), which was higher compared with C2C12 cells (1.6-fold).

#### Substrate oxidation.

**Fig. 5.**
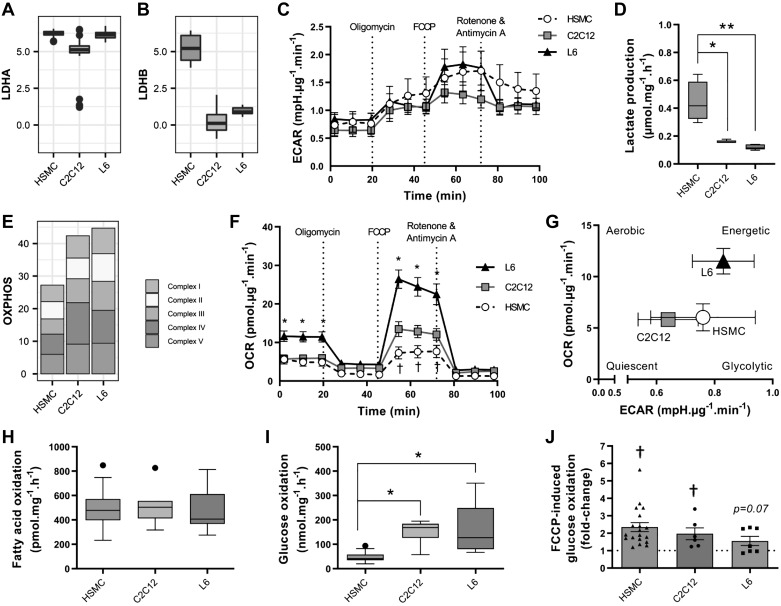
Oxygen consumption and substrate use in L6, C2C12, and human primary myotubes. *A* and *B*: mRNA expression of lactate dehydrogenase subunits (*LDHA*, *LDHB*). *C*: extracellular acidification rate (ECAR) during a Mito Stress Test measured with Seahorse technology. Means ± SE; *n* totals are as follows: HSMC: *n* = 13 replicates from 8 independent donors, C2C12: *n* = 7 independent experiments, L6: *n* = 8 independent experiments, two-way ANOVA with Tukey’s multiple comparison. *D*: lactate accumulation in the extracellular medium measured as described in methods. Box-and-whisker Tukey plot; *n* totals are as follows: HSMC: *n* = 5 replicates from 4 independent donors, C2C12: *n* = 5 independent experiments, L6: *n* = 5 independent experiments, one-way ANOVA with Tukey’s multiple comparison, *P < 0.05, **P < 0.01. *E*: mRNA expression of mitochondrial complexes. *F*: oxygen consumption rate (OCR) during a Mito Stress Test measured with Seahorse technology. Means ± SE; *n* totals are as follows: HSMC: *n* = 13 replicates from 8 independent donors, C2C12: *n* = 7 independent experiments, L6: *n* = 8 independent experiments, two-way ANOVA with Tukey’s multiple comparison, *P < 0.05 compared with both C2C12 and human skeletal muscle cells (HSMC), †*P* < 0.05 compared with C2C12 and L6. *G*: energy plot from the Mito Stress Test. *H*: unstimulated fatty acid oxidation, measured by the oxidation of [^3^H]palmitate into H_2_O. Box-and-whisker Tukey plot; *n* totals are as follows: HSMC: *n* = 37 replicates from 12 independent donors, C2C12: *n* = 11 independent experiments, L6: *n* = 17 independent experiments, one-way ANOVA with Tukey’s multiple comparison. *I*: basal glucose oxidation as measured by the oxidation of [^14^C]glucose into CO_2_. Box-and-whisker Tukey plot; *n* totals are as follows: HSMC: *n* = 19 replicates from 12 independent donors, C2C12: *n* = 6 independent experiments, L6: *n* = 8 independent experiments, one-way ANOVA with Tukey’s multiple comparison, *P < 0.05. *J*: carbonyl cyanide-4-(trifluoromethoxy)phenylhydrazone (FCCP)-stimulated [^14^C]glucose oxidation. Means ± SE and individual data points; *n* totals are as follows: HSMC: *n* = 19 replicates from 12 independent donors, C2C12: *n* = 6 independent experiments, L6: *n* = 7 independent experiments, one-way ANOVA with Tukey’s multiple comparison, †*P* < 0.05 effect of FCCP vs. unstimulated. OXPHOS, oxidative phosphorylation.

Skeletal muscle is a metabolically flexible organ, able to switch from carbohydrate to fatty acid oxidation depending on whole-body energy demands. Under in vitro conditions, cells rely heavily on anaerobic glycolysis, even in the presence of oxygen and fatty acids, in a manner similar to the Warburg effect observed in cancer cells (52). L6, C2C12, and HSMC cells exhibited similar mRNA levels of enzymes of the glycolysis pathway (Supplemental Table S2; see https://doi.org/10.5281/zenodo.1246757), but HSMC had higher mRNA expression of lactate dehydrogenase B (Fig. 5, *A* and *B*). The extracellular acidification rate (ECAR) was similar between the models during the 100 min of the Seahorse assay (Fig. 5*C*), but accumulation of lactate in the medium over 24 h was higher in HSMC compared with L6 and C2C12 cells (Fig. 5*D*). The mRNA levels of proteins involved in the mitochondrial respiratory chain were higher in L6 compared with C2C12 and HSMC cells (Fig. 5*E*), which translated into higher rates of oxygen consumption in L6 cells (Fig. 5*F*). Overall, the L6 cells had higher metabolic rates and primarily relied on aerobic metabolism, while C2C12 and HSMC cells relied on anaerobic glycolysis (Fig. 5*G*).

Differences in oxygen consumption may be attributed to the use of either glucose or fatty acids by the mitochondria. To derive substrate preference, we separately measured glucose and fatty acid oxidation using radiolabeled substrates. Fatty acid oxidation at baseline was similar in the three models (Fig. 5*H*). Baseline glucose oxidation was lowest in HSMC cells (Fig. 5*I*). The uncoupling agent trifluoromethoxy carbonylcyanide phenylhydrazone increased glucose oxidation to a similar extent in all models (FCCP; Fig. 5*J*).

#### Structural and contractile differences.

**Fig. 6.**
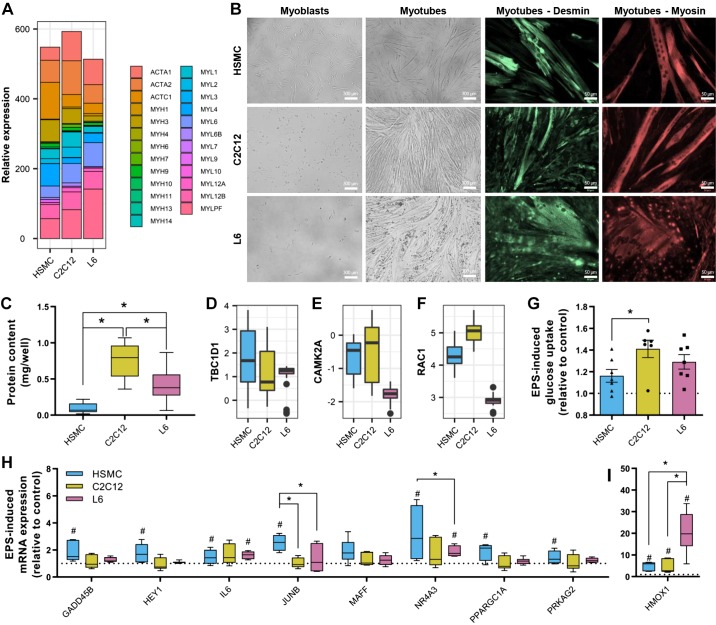
Response to electrical pulse stimulation (EPS) in L6, C2C12, and human primary myotubes. *A*: mRNA levels of myosin and actin isoforms. *B*: representative images of myotubes during proliferation and after complete differentiation. Staining for desmin and myosin was performed using specific antibodies as described in methods. Representative images are shown for each staining. *C*: protein content per well measured by bicinchoninic acid assay. Box-and-whisker Tukey plot; *n* totals are as follows: HSMC: *n* = 11 replicates from 11 independent donors, C2C12: *n* = 7 independent experiments, L6: *n* = 6 independent experiments, Kruskal-Wallis test with Dunn’s multiple comparison. *D–F*: mRNA levels of the GTPase-activating protein for Rab family protein *TBC1D1*, Ca^2+^/calmodulin-dependent protein kinase II (*CAMK2A*), and *RAC1*. *G*: electrical pulse stimulation-induced glucose uptake measured using 2-[1,2-^3^H]deoxy-d-glucose. Mean ± SE and individual data points: *n* totals are as follows: HSMC: *n* = 7 replicates from 7 independent donors, C2C12: *n* = 6 independent experiments, L6: *n* = 7 independent experiments, one-way ANOVA with Tukey’s multiple comparison. *H* and *I*: mRNA expression of exercise-responsive genes measured by quantitative PCR. Box-and-whisker Tukey plot; *n* totals are as follows: HSMC: *n* = 5 replicates from 5 independent donors, C2C12: *n* = 6 independent experiments, L6: *n* = 6 independent experiments, two-way ANOVA with Fisher’s least-significant difference comparison. #*P* < 0.05, significant response to EPS. *P < 0.05. HSMC, human skeletal muscle cells.

HSMC, L6 and C2C12 cells exhibited striking differences in mRNA profiles of actin and myosin isoforms, with *ACTC1*, *MYH1*, and *MYL2* enriched in human myotubes, whereas *ACTA2* and *MYL1* mRNA was highest in C2C12 cells, and *MYLPF* mRNA was highest in L6 myotubes (Fig. 6*A*). After differentiation, C2C12 and HSMC cultures exhibited long, thin myotubes that were uniformly aligned, while L6 cultures formed a disorganized network of thick myotubes (Fig. 6*B*). Desmin staining was similar in all three cell types, but HSMC and C2C12 exhibit a clearer expression of myosin in fully differentiated myotubes (Fig. 6*B*). Overall, a monolayer of C2C12 myotubes had the highest protein content compared with L6 and HSMC cells (Fig. 6*C*).

Electrical pulse stimulation leads to sarcomere reorganization and actual contraction of C2C12 myotubes (15), and to a lesser extent, primary human myotubes (23) can readily be observed. L6 myotubes do not, however, exhibit any obvious contractile activity (26). Contraction-induced glucose uptake in skeletal muscle depends on the activation of Ca^2+^/calmodulin-dependent protein kinase II (CamKII), Rac1, and the GTPase-activating protein for the Rab family protein TBC1D1 (46). *TBC1D1* is similarly abundant in all three cell models (Fig. 6*D*), but L6 myotubes had lower amounts of *CAMK2A* and *RAC1* mRNA (Fig. 6, *E* and *F*). Despite these differences, electrical pulse stimulation increased glucose uptake in all cell models with rates of EPS-stimulated glucose uptake higher in C2C12 versus HSMC (Fig. 6*G*). Exercise-induced gene expression can be reproduced with EPS in vitro by measuring specific exercise-responsive genes (13). EPS-induced transcription differed slightly across the models; HSMC had higher expression of *JUNB* and *NR4A3* mRNA (Fig. 6*H*), whereas *HMOX1* mRNA induction was greater in L6 compared with C2C12 and HSMC cells in response to EPS (Fig. 6*I*).

## DISCUSSION

Cellular models are vital tools to study biology in controlled environments, providing mechanistic insight into the molecular pathways and cellular responses involved in healthy and pathological states. Mouse C2C12, rat L6, and human primary myotubes are widely used in vitro models of skeletal muscle, each with strengths and limitations that have been thoroughly characterized in the present study. We identified important transcriptomic and metabolic differences between C2C12, L6, and human primary cells that arise from both species and intrinsic divergence across cell models.

### 

#### C2C12 and human primary myotubes are enriched in mRNA of genes encoding contractile proteins.

Compared with L6 myotubes, mouse C2C12 and human primary myotubes are enriched in genes linked to muscle development and contraction, which is associated with elongated myotubes and a higher abundance of myosin. Primary human cells exhibit the highest levels of *MYH7* and *MYL2*, proteins associated with type I fibers, while C2C12 myotubes have the highest expression of *MYH1* and *MYH4*, proteins associated with type II (glycolytic) fibers (41). Fiber-type composition varies greatly across species, with rodent skeletal muscle consisting predominantly of 2X and 2B fibers and human skeletal muscle consisting of type 1 and 2A fibers (41). Thus the rodent origin of C2C12 and L6 cells may contribute to differences in myosin composition compared with HSMC cells. C2C12 cells have been intensively used to study effects of in vitro contraction on gene expression and metabolism, using either electrical pulse stimulation or manipulating calcium signaling (18, 31, 32, 42). The higher abundance of contractile proteins in C2C12 cells, as well as the better response to EPS-induced glucose uptake, confirms that they are a superior model for contraction studies than HSMC and L6 cells. However, the mRNA level of genes encoding contractile proteins in all cell models was much lower than fully developed adult tissue (Supplemental Table S2; see https://doi.org/10.5281/zenodo.1246757). The precise mechanism for this difference is unknown but may be related to the lack of innervation, vascularization, hormonal factors, and cellular crosstalk in cells, as these factors are required for skeletal muscle development and repair (38). Denervation in animal models or spinal cord injury in humans leads to skeletal muscle atrophy and a sharp decrease in contractile protein content and metabolism (24), but skeletal muscle satellite cells retain their differentiation ability (39). Coculture with neurons in vitro promotes differentiation of muscle cells into striated fibers that are able to contract spontaneously (3), demonstrating the importance of innervation in skeletal muscle differentiation. The absence of innervation in cultured cells likely keeps the myocyte in an immature state.

#### L6 myotubes have the highest insulin-induced glucose uptake.

The coordinated action of multiple tissues is required for whole-body fuel homeostasis, but insulin-mediated blood glucose clearance is primarily accounted for by skeletal muscle (49). Maintenance of skeletal muscle insulin sensitivity and metabolic flexibility, through healthy diet and exercise, prevents or delays the development of type 2 diabetes (9). Thus exercise is an effective therapeutic intervention to combat metabolic diseases, thereby placing skeletal muscle biology at the core of metabolic research. Our transcriptomic profiling revealed that L6 myotubes have low mRNA expression of the glucose transporters GLUT1 and GLUT3 but the highest expression of GLUT4. In vivo, GLUT1 and GLUT3 are ubiquitously expressed and responsible for baseline glucose uptake. This altered pattern of GLUT expression in cultured cells versus muscle tissue is not an unexpected finding, since GLUT3 is abundant in human fetal muscle, which shares similarities with muscle cells in culture (43). In human adult muscle, GLUT3 is enriched in slow-twitch muscle fibers compared with fast-twitch fibers (44). The distribution of *SLC2A3* (*GLUT3*) mRNA in mouse tissue is more restricted than in human tissues, with expression essentially confined to the brain (30). These data align with the reduced GLUT3 mRNA within C2C12 and L6 myotubes compared with HSMC cells. Insulin-stimulated glucose uptake depends on glucose transporter 4 (GLUT4), which is most abundant in mature mammalian adipose, and muscle cells (19, 54). In this regard, L6 myotubes have a pattern of GLUT expression closer to fully differentiated mammalian muscle, with high levels of GLUT4 and relatively low expression of GLUT1 and GLUT3. This pattern of GLUT expression is consistent with our observation that L6 myotubes have the lowest rate of basal glucose uptake but the highest insulin-induced response, making them a valuable tool for metabolic studies.

In muscle cells in vivo, most of the glucose sequestered during rest is stored in the form of glycogen. This process is thought to be dependent on the inhibitory phosphorylation of GSK3 by PKB/Akt downstream of the insulin receptor (33). In response to insulin, primary human myotubes have the highest rate of glycogen synthesis (2.1-fold). Insulin-induced glycogen synthesis in L6 myotubes increased by 1.8-fold, despite the fact that GSK3 is lowly expressed, consistent with observations of similar glycogen content in mice with inactive GSK3 compared with wild-type mice (7). Collectively, these results suggest that allosteric regulation of glycogen synthase by glucose-6-phosphate plays a critical role in the insulin-stimulated synthesis of glycogen in L6 myotubes, as reported for mouse skeletal muscle in vivo (8).

#### Human primary myotubes have the lowest oxidative capacity.

Skeletal muscle fibers are rich in mitochondria, the activity of which is required to sustain ATP production via oxidative phosphorylation. Within skeletal muscle tissue, the oxidative fibers have particularly high mitochondrial content, and the capacity for oxidative phosphorylation provides resistance to fatigue (28). From the cell types tested in this study, HSMCs have the highest mRNA expression of GLUT3, citrate synthase (Supplemental Table S2; see https://doi.org/10.5281/zenodo.1246757), and myosin heavy chains associated with type I fibers (41), suggesting that these cells better represent adult oxidative fibers compared with L6 and C2C12 cells. However, the oxygen consumption rate of HSMC cells is paradoxically lower than all other muscle cell types tested. Reduced mitochondrial content and activity are observed in type 2 diabetes, physical inactivity, and aging in vivo (16, 21, 48), suggesting that HSMC cells in vitro possess lower mitochondrial content and/or dysfunctional mitochondria compared with muscle cells in their physiological environment.

Oxygen consumption was the highest in L6 myotubes compared with C2C12 and HSMC cells, despite our finding that these cells have the lowest basal glucose uptake. Curiously, baseline glucose uptake, substrate oxidation, and oxygen consumption were not correlated with each other. Fatty acid oxidation rates were similar in the three cell models, but glucose oxidation was substantially different. The glucose oxidation assay measures CO_2_ released from glucose, making it a proxy of TCA cycle activity, whereas the lipid oxidation and Seahorse assays estimate mitochondrial oxidative phosphorylation. These metabolic assays suggest that L6 cells have the most robust coupling between glucose uptake and oxidative phosphorylation. HSMCs divert glucose to glycolysis, while C2C12 likely diverts glucose from the TCA cycle to amino acid synthesis (5, 40). This finding may be related to the relatively high abundance of mRNA coding for contractile proteins in C2C12 and HSMC cells with L6 cells, which would require enhanced TCA cycle activity to support amino acid synthesis. Overall, our data demonstrate that L6, C2C12 and HSMC cells have a fundamentally different metabolism and may divert substrates into selective pathways based on the need for protein synthesis or energy.

#### Conclusion.

**Fig. 7.**
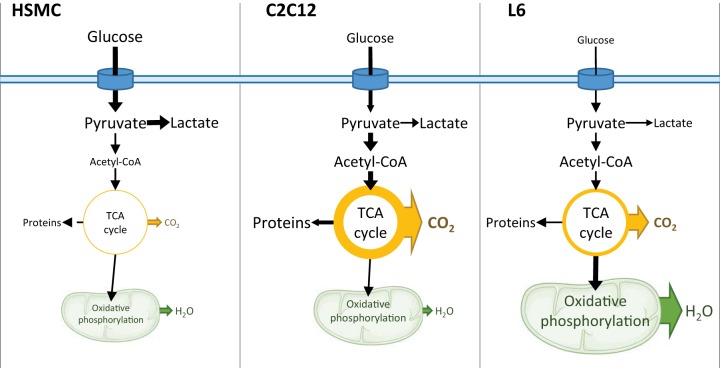
Schematic representation of C2C12, L6, and human primary myotube metabolic pathways. Fatty acid oxidation was similar in all three cell models; therefore changes in glucose metabolism between the cell types may explain the differences in metabolism across models. Human primary myotubes have the highest baseline glucose uptake and lactate production, suggesting that these cells rely mostly on glycolysis for energy production. On the other end of the spectrum, L6 myotubes have a relatively lower baseline glucose uptake, but these cells oxidize most of the intracellular glucose in the mitochondria. C2C12 cells have moderate rates of glucose uptake, with intracellular glucose directed toward the TCA cycle. Oxidative phosphorylation and TCA coupling to oxidative phosphorylation are greater in L6. The thickness of the arrows and the size of text represent the intensity of the corresponding pathways compared with the other models. HSMC, human skeletal muscle cells.

Our study provides experimental evidence that rat L6, mouse C2C12, and human primary myotubes exhibit profoundly different transcriptomic profiles and metabolic behaviors (Fig. 7). L6 myotubes are more suitable for studies of glucose metabolism and mitochondrial function, while C2C12 and primary human myotubes resemble differentiated muscle tissue in terms of myosin content and glycogen storage and may be more appropriate for studies of exercise/stress responses. These cell-specific and tissue-specific responses are dependent on the unique transcriptomic profile of each system. This transcriptomic plasticity is presumably conferred by the transformed nature of L6 and C2C12, the variability in human donors for the HSMC, and epigenetics that are influenced by many factors, such as differences in batches of fetal bovine serum, growth and counting of passages, and the heterogeneous storage conditions of cells over several decades between laboratories. Our study establishes the utility of each cellular system to model adult skeletal muscle and provides an evidence-based rationale to consider when selecting either L6, C2C12, or human primary cells to explore developmental and metabolic processes in skeletal muscle.

## GRANTS

The authors are supported by grants from the Novo Nordisk Foundation (NNF14OC0011493, NNF17OC0030088, and NNF14OC0009941), Swedish Diabetes Foundation (DIA 2018-357 and DIA2018-336), Swedish Research Council (2015-00165 and 2018-02389), Strategic Research Program in Diabetes at Karolinska Institutet (2009-1068), Stockholm County Council (SLL20170159), Swedish Research Council for Sport Science (P2018-0097), and European Foundation for the Study of Diabetes (EFSD) European Research Programme on New Targets for Type 2 Diabetes, supported by an educational research grant from MSD. L. Dollet was supported by a Novo Nordisk postdoctoral fellowship run in partnership with Karolinska Institutet. B. M. Gabriel was supported by a fellowship from the Wenner-Gren Foundation (Sweden). N. J. Pillon and L. Sardón Puig were supported by Marie Skłodowska-Curie Actions (European Commission, 704978 and 675610) and N. J. Pillon, grants from the Sigurd och Elsa Goljes Minne and Lars Hiertas Minne Foundations (Sweden). L. Sardón Puig was supported by KID funding from Karolinska Institutet (2-3591/2014).

## DISCLOSURES

No conflicts of interest, financial or otherwise, are declared by the authors.

## AUTHOR CONTRIBUTIONS

A.M.A. and N.J.P. conceived and designed research; A.M.A., L.S.P., J.A.B.S., B.M.G., M.S., L.D., A.V.C., and N.J.P. performed experiments; A.M.A., L.S.P., J.A.B.S., B.M.G., M.S., L.D., A.V.C., and N.J.P. analyzed data; A.M.A., L.S.P., J.A.B.S., B.M.G., M.S., L.D., A.V.C., A.K., J.R.Z., and N.J.P. interpreted results of experiments; A.M.A. and N.J.P. prepared figures; A.M.A. and N.J.P. drafted manuscript; A.M.A., L.S.P., J.A.B.S., B.M.G., M.S., L.D., A.V.C., A.K., J.R.Z., and N.J.P. edited and revised manuscript; A.M.A., L.S.P., J.A.B.S., B.M.G., M.S., L.D., A.V.C., A.K., J.R.Z., and N.J.P. approved final version of manuscript.
